# Using hospitalization for ambulatory care sensitive conditions to measure access to primary health care: an application of spatial structural equation modeling

**DOI:** 10.1186/1476-072X-8-51

**Published:** 2009-08-28

**Authors:** Md Monir Hossain, James N Laditka

**Affiliations:** 1 Biostatistics, Epidemiology and Research Design (BERD) Core, Center for Clinical and Translational Sciences, The University of Texas Health Science Center at Houston, UT Professional Building, Room 1100.25, 6410 Fannin Street, Houston, TX, 77030, USA; 2 Department of Public Health Sciences, University of North Carolina at Charlotte, Charlotte, NC 28223, USA

## Abstract

**Background:**

In data commonly used for health services research, a number of relevant variables are unobservable. These include population lifestyle and socio-economic status, physician practice behaviors, population tendency to use health care resources, and disease prevalence. These variables may be considered latent constructs of many observed variables. Using health care data from South Carolina, we show an application of spatial structural equation modeling to identify how these latent constructs are associated with access to primary health care, as measured by hospitalizations for ambulatory care sensitive conditions. We applied the confirmatory factor analysis approach, using the Bayesian paradigm, to identify the spatial distribution of these latent factors. We then applied cluster detection tools to identify counties that have a higher probability of hospitalization for each of the twelve adult ambulatory care sensitive conditions, using a multivariate approach that incorporated the correlation structure among the ambulatory care sensitive conditions into the model.

**Results:**

For the South Carolina population ages 18 and over, we found that counties with high rates of emergency department visits also had less access to primary health care. We also observed that in those counties there are no community health centers.

**Conclusion:**

Locating such clusters will be useful to health services researchers and health policy makers; doing so enables targeted policy interventions to efficiently improve access to primary care.

## Background

Hospitalization for Ambulatory Care Sensitive Conditions (ACSCs) is a health care indicator that has been used extensively to study the accessibility of health care (AHC). The measure has been endorsed by the United States Institute of Medicine [1] and the Agency for Healthcare Research and Quality [2]. Accessible and reasonably effective primary health care can potentially reduce the risk of hospitalization for ACSCs. Thus, a higher rate of hospital admissions for ACSCs in an area may provide evidence of underlying problems with population access to health care. The theory underlying the ACSC indicator has been supported empirically; lower availability of primary care has been associated with higher rates of ACSC admissions [3-6]. Mobley et al. [7] showed the spatial distribution of ACSC admissions for the entire United States and observed clustering. This result suggested geographic variation of access to health care. Spatial analysis provides a tool to control this variation, thereby improving estimates of associations between ACSCs and other factors.

One notable reason for the usefulness of the ACSC indicator is that it is often applied using readily available population rates of hospitalization. Models that estimate the risk of ACSC admissions can account for a range of factors in addition to access to health care, such as population lifestyle, physician practice behaviors, population tendency to use health care resources, and disease prevalence [8-10]. Using administrative health care data most commonly used to study hospitalizations for ACSCs, many of these factors are not measurable quantities, i.e., they are latent. The complex relationships among these factors have received little attention [11]. One way to conceptualize their relationship with access to health care is as a complex latent construct of observable and potentially observable variables, i.e. the ACSC hospitalization rate and other variables that are often unobservable in a given data set. Because of the unobservable nature of many factors, structural equation modeling may be the best way to understand the intricate relationships among these factors.

We are specifically interested in applying the confirmatory factor analysis (CFA) approach in the context of structural equation modeling to identify how population lifestyle, physician practice behaviors, population tendency to use health care resources, and disease prevalence are associated with access to health care. In CFA, the structure for the latent variables is prespecified and, thus, determines how the model parameters should be constrained. Here, our primary purpose is to model the relationships among the multiple latent variables, whereas we are not interested in the distributional properties of the latent variables. This enables us to standardize the manifest variables that are related to exogenous factors to have zero means and unit variances. In addition, some of the regression coefficient parameters in the measurement models will be constrained according to a prespecified structure.

Structural equation models are well established for multivariate Gaussian response variables [12]. Generalization to the exponential family of distributions is more recent [13]. For manifest variables that are spatially referenced, structural equation models have been proposed for continuous variables in [14,15]. Liu et al. [16] and Wang and Wall [17] generalize this application to the exponential family of distributions. Congdon et al. [18] extended the generalized spatial structure equation models to incorporate spatially-structured and unstructured random effects at the measurement level.

## The conceptual model for access to health care (AHC)

**Figure 1:**
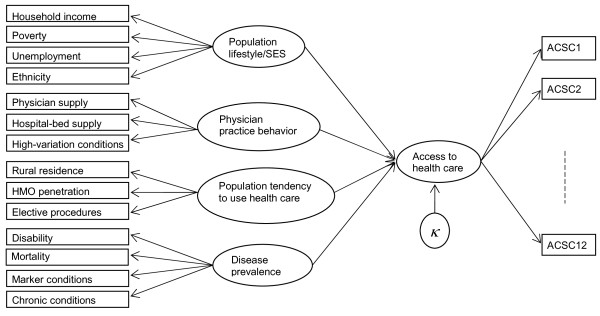
**Conceptual model to assess the underlying factor, access to health care**.

Researchers have rarely noted that high ACSC admission rates at a geographical unit of measurement (e.g. county or zip code) may not exclusively indicate inadequate access to primary health care. They may also indicate unhealthful population lifestyles, physician practice behaviors that vary among geographic areas due to differences in training or the cultures of local medical communities, the tendency of the area population to use preventive health care, and/or high rates of disease [8,9,19]. These facts challenge the use of ACSCs as a measure of AHC, unless the analysis adjusts for such factors. This framework for understanding the dynamics of health care access resulted in the development of a conceptual model (Figure 1), where ovals indicate underlying factors, rectangles indicate observed variables, and an arrow with a solid line indicates the direction of flow of information.

A number of alternative models can also be conceptualized along these lines. Our purpose in the present study is not to identify a "perfect" theoretical model of ACSC hospitalization or to include all observable variables that might be suggested for such a model, but rather to illustrate the usefulness of a statistical method for identifying areas with poor access to health care. Nonetheless, the model presented in this study should be adequate to suggest geographical areas where further research should be concentrated to reduce potential barriers to the accessibility of primary health care. The methods used in this paper could be usefully applied to other geographical areas as well as a wide variety of questions in public health and health services research.

Instead of modeling hospital admissions for ACSCs as a single measure of health care access, we propose to model twelve adult ACSCs individually and adopt a multivariate approach. To our knowledge this is the first work that treats ACSCs as a multivariate concept, rather than a univariate one, in a spatial factor analytic approach. These twelve manifest variables represent ACSCs: short-term diabetes complications, long term diabetes complications, uncontrolled diabetes, lower extremity amputation in individuals with diabetes, adult asthma, hypertension, dehydration, urinary tract infection (UTI), bacterial pneumonia, angina without procedure, chronic obstructive pulmonary disease (COPD) and congestive heart failure (CHF). In Figure 1, these twelve ACSCs correspond to ACSC1 through ACSC12. The multivariate approach will allow us to incorporate the correlation structure among the ACSCs into the model. This is useful because some of the ACSCs share common comorbidities, and others share common behavioral risk factors. Aggregating all ACSCs into a single variable would lose this information, introducing potentially substantial bias into the estimates. The latter approach has been used in almost all previous research that relies on the ACSC indicator. Thus, the present method may provide a notable opportunity to improve research that relies on this widely-used indicator.

The above conceptual model will be validated at the county level by a multivariate spatial factor analysis. The analysis will then potentially involve two confounded dimensions of dependency: between different variables and between different spatial locations. The research question that we will address is how population lifestyle, physician practice behaviors, population tendency to use health care resources, and disease prevalence are associated with a common spatial factor underlying ACSC admissions. We will look for a regression relationship among these variables by a confirmatory factor analysis approach, where the factor underlying the twelve ACSC admissions is the dependent variable, and population lifestyle, physician practice behaviors, population tendency to use health care resources, and disease prevalence are independent variables. We assume that the independent variables and the common factor (access to care) underlying the twelve ACSC admission types are complex latent constructs rather than measurable quantities. Structural equation modeling treats these constructs as underlying latent factors and finds their relationships through the manifest variables used to measure them.

### Manifest variables

The manifest variables are the observed data used to measure the latent factors and examine the causal connections between these factors. In our model, all of the manifest variables are measured at the county level.

Four variables are used to measure population lifestyle or socio-economic status (SES): household income, percentage of the population below the poverty level, unemployment rate per 1000 population, and ethnicity. The measure of household income is the median household income. Ethnicity is measured by the percentage of the population that is African-American. This ethnicity definition is reasonable in the South Carolina context; a large majority of residents are either African American or non-Hispanic white, both statewide and within each county, and the proportion that is African American is substantial in every county. Other socio-economic variables, e.g., education level (measured by years of educational attainment), could be included among the measures for this latent factor.

Three variables measure physician practice behavior: physician supply per 1000 population, hospital beds per 1000 population, and hospitalizations for high variation conditions per 1000 population. The first two measures can affect practice patterns due to supplier-induced demand; when the supply of physicians or hospital beds grows to a level where the individual physician or hospital must compete to maintain income, the likelihood of supplier-induced demand may rise [9]. High variation conditions are those for which hospitalizations vary greatly among areas [8,20]. Hospitalization for these conditions involves physician discretion in treatment options; high rates of hospitalization for these conditions in a county may suggest underlying problems in medical decision making or differences associated with physician training or local practice cultures. We use the list of medical DRGs for high-variation conditions provided by the Dartmouth Atlas of Health Care [21].

Three variables are used to measure population tendency to use health care: rural residence, the penetration of Health Maintenance Organizations (HMOs) in the area, and elective procedures. Rural residence is a proxy measure of travel time and other barriers to accessing physicians. This can be conceptualized as an ordinal variable, with 10 categories of rurality. One previous study used an ordinal definition of rurality of this sort, and found a notable gradient of hospitalization across levels of rurality [22]. HMO penetration rate influences physician practice behavior. Physicians in areas with high HMO penetration tend to practice in a more preventative way (according to the HMO guidelines) than physicians in low HMO penetration areas, even when the patient is covered by fee-for-service insurance [23]. Elective procedures are planned, non-emergency surgical procedures. They may be either medically required (e.g., cataract surgery) or optional (e.g., breast augmentation or implant) surgery. Elective surgeries may extend life or improve the quality of life physically and/or psychologically. However, they nonetheless provide a measure of population tendency to use health care since rates of such surgeries vary notably among both small areas and large geographical regions.

Four variables measure disease prevalence: disabled population per 1000, mortality per 1000 population, hospitalizations for marker conditions per 1000 population, and hospitalizations for chronic conditions per 1000 population. Disability is measured by the number of people who receive Social Security benefits for disability. Instead of a blanket 'mortality' measure, we use mortality for liver disease as a measure of excessive alcohol consumption. We also use mortality for heart disease, COPD, and diabetes [5]; the latter three mortality measures are for ACSCs. The rationale for using these measures is to control for disease severity, which is presumably associated with mortality for these diseases. Death rates for these diseases may also indicate health care access barriers; areas with inadequate access may have higher death rates. Thus, including these death rates may over-adjust ACSC rates, providing conservative estimates. Hospitalizations for marker conditions are taken to be measures of population health. Marker conditions include hospitalizations for appendicitis with appendectomy, acute myocardial infarction (AMI), gastrointestinal obstruction and hip fracture. Hospitalizations for these conditions are not typically associated with physician supply, physician practice patterns, or related variables. Another important predictor for population health is the proportion of the population with chronic conditions. For a list of these conditions, we used the Chronic Condition Data Warehouse User Manual [24].

**Figure 2:**
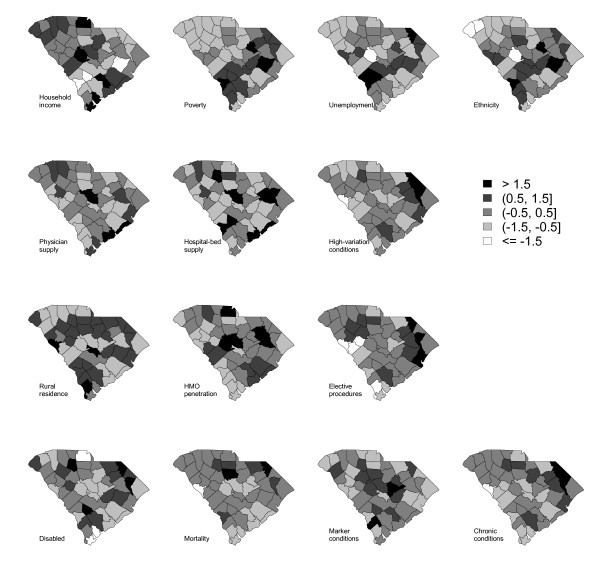
**Thematic maps of the observed variables for underlying factors population lifestyle/SES (first row), physician practice behavior (second row), population tendency to use health care (third row) and disease prevalence (fourth row)**.

Figure 2 displays thematic maps of these manifest variables that are used for constructing the exogenous variables. In this display, all of these manifest variables are transformed to have mean zero and standard deviation one. The first row shows the four manifest variables that measure population lifestyle/SES. The map for household income depicts an opposite pattern from the maps for the other three variables. The second row shows the three manifest variables that measure physician practice behavior. These three maps do not show any common pattern. The third row shows the three manifest variables that measure population tendency to use health care. The map for the HMO penetration rate shows an opposite pattern from the maps for the other two variables. The fourth row shows the four manifest variables that measure disease prevalence. These four maps show similar patterns.

## Statistical models for access to health care

In the statistical model corresponding to the conceptual model for AHC, we have used the generalized spatial structural equation models proposed by Liu et al. [16] and Wang and Wall [17]. It is a two-level hierarchical model; the first-level is a measurement model that can accommodate any distributions from the exponential family. The second-level is a structural equation model.

In the example below, we illustrate the implementation details of this model for the modeling of AHC, the use of cluster detection tools to find the counties with notable access risks for each type of ACSC admissions, and use of a model selection criterion to validate the model.

### Generalized spatial structural equation models for AHC

In the above conceptual model, the total number of latent factors is five (i.e., *q *= 5). Among them, one is an endogenous variable (i.e., *q*_1 _= 1) and four are exogenous variables (i.e., *q*_2 _= 4). The total number of manifest variables is twenty-six, for which *p*_1 _= 12, *p*_2 _= 4, *p*_3 _= 3, *p*_4 _= 3 and *p*_5 _= 4.

The observed number of hospital visits for ACSC1,...,ACSC12 are , respectively, manifest the first factor (access to health care). We assume a Poisson distribution for each  with mean , where *j *= 1,...,12. For the other observed data,  manifest the second factor (population lifestyle/SES),  manifest the third factor (physician practice behavior),  manifest the fourth factor (population tendency to use health care), and  manifest the fifth factor (disease prevalence). These observed variables for exogenous factors are standardized to have mean zero and standard deviation one, such that the original scale will have no more influence. These transformed variables are then fitted to normal distributions. Thus, the measurement models are(1)

and the joint mean structure with the constraint values to some factor loadings is(2)

where, *E*_*ij *_is the expected count of hospitalization for *j *th ACSC in *i *th county.

The structural equation model for the relationship between latent factors for county *i *is(3)

where *κ*_*i *_has a univariate proper conditional autoregressive (CAR) distribution, defined by(11)

where, ,  and {*κ*_-*i*_} is the set of *κ*'s who share the common boundary with the *i *th region.

The joint distribution for **f**_2*i *_is defined by the linear model of coregionalization method [25-27] as(5)

where the **u**_*j*_'s have proper CAR distributions as defined in (4).

#### Prior specifications and Posterior distribution

Under the Bayesian paradigm, it is essential to set a prior distribution for each parameter to be estimated. Five factor loadings in (2), , , ,  and , are set to one in order to remove scale uncertainty. Mean-zero normal uninformative prior distributions are assigned to other loading factors  (*j *= 1,..., *p*_*k*-1_, *k *= 1,...,5) in (2), and to the *χ *'s in the structural equation model in (3). Uninformative inverse-gamma priors are assigned to the scale parameters , ,  and  in (1), *σ*_*κ *_in (4), and *a*_1_, *a*_3_, *a*_6 _and *a*_10 _in (5). Uniform distributions with the values 0 and 1 are considered prior distributions for the spatial correlation parameters *ρ*_*κ *_in (3), and  in the distribution of **u**_*j *_in (5).

Let **Γ **be the vector that contains all the unknown parameters, **O **be a vector of order 26*n *of all the manifest variables, **f**_1 _be a vector of order *n *of endogenous factors and **f**_2 _be a vector of order 4*n *of exogenous factors. The joint posterior distribution of all the unknowns is defined as

where the elements of *a*_*κ *_are the parameters for the distribution of ***κ***, the elements of  are the parameters for the distribution of **u**_2_, and *p*(**Γ**) is the product of each prior distribution.

### Spatial cluster detection

In the measurement model, the Poisson models for the twelve adult ACSCs are given as , where  is the log-relative risk for *j *th ACSC and *j *= 1,...,12. It is of interest to find the counties where the rate of hospitalization is high for specific ACSCs, as this has clinical relevance for the design of targeted interventions to improve the medical management of those conditions.

In order to find these counties, we apply a cluster detection tool that is developed in Hossain and Lawson [28] for spatial data. A cluster is a geographically and/or temporally bounded group of occurrences of sufficient size and concentration that it is unlikely to have occurred by chance [29]. Some cluster detection tools proposed in Hossain and Lawson [28] are based on neighborhood information, with the belief that clustering could have spatial integrity, and some are based on error rates (e.g., misclassification rate, mean square error).

From the maps, we will be interested to identify the counties with excess risks for ACSC hospitalizations, i.e., clusters. We first calculate the *posterior exceedence probability *(PEP), i.e., the probability of ACSC specific relative risk estimates exceeding a given threshold value. This is often used to assess localized single region hot-spot clusters. It is assumed that estimates of  are available from posterior sampling. The exceedence probability of the sampled  can be computed as , where  is the estimate of  for the *g*th sample value from converged posterior sampling output, *G *is the posterior sample size and *c *is a factor-specific threshold value. The choice of a value for *c*, which is critical, can be made according to the study objectives. One choice could be the value one. This probability estimate is commonly used to provide evidence of notable excess risk in individual counties [30]. Notable excess risk can be regarded as a criterion for identifying 'hot-spot' clusters.

We could use PEP to examine a single county. However, it may be reasonable to believe that clustering should have some spatial integrity, in which case criteria that also examine county-level neighborhoods around points could be useful. Define a set, {*q*_*ijk*_; *k *= 0,1,..., *n*_*i*_}, of first-order neighbor *q *values of the *i *th county, *j *th ACSC in *k *th neighboring county, where *n*_*i *_is the number of first-order neighbors of the *i *th county that share a common geographical boundary, and *q*_*ij*0 _is the *q *value of the *i *th county and the *j*th ACSC.

A local measure *R*_*ij*_, can be proposed as

to calculate the proportion having exceedence probability greater than 0.95 based on the first-order neighbors. The first indicator function in the right hand side of the above equation, *I*(*q*_*ij *_> 0.95), is to ensure that only counties having excess risk are used to find clusters. The measure *R*_*ij *_shows the grouping of excess risk regions where the posterior probability of excess risk is greater than 0.95. In this way, a surface of *R*_*ij *_can be derived, which will give evidence of clusters of excess risk and can be used to detect unique clusters. Note that there is a trade off between the choice of *c *and the chosen critical probability value (here defined as 0.95). Higher values of *c *will lead to fewer regions signaling, while lower critical probability values will admit more regions.

### Model estimation and validation

To estimate the models, we used software written by the first author in the WinBUGS programming language [31]. The computer code used for this research is available from the first author on request. The maps were produced in R [32]. The reported model results are the posterior mean over 20,000 MCMC samples after a burn-in period of 1,000,000 samples for each estimated unknown parameter. Because the model is complex, this relatively long burn-in period was used to ensure convergence. We also checked the density plot and the trace plot of each parameter.

To validate our conceptual model, we will consider a number of alternative models based on spatial and/or independent effects at different hierarchical levels; the best model will be chosen by a model selection criterion. As an aid to model selection we use the deviance information criterion (DIC) [33]. In a Bayesian paradigm, DIC seems the most appropriate model selection criterion since it exploits the deviance statistics of GLM as a measure of goodness-of-fit, and then penalizes it by the effective number of parameters. Another possibility is to use the mean square prediction error (MSPE). The MSPE is the posterior predictive loss under the squared error loss function as described in Gelfand and Ghosh [34]. The MSPE is the mean squared difference between the observed and the predicted values of the outcome variable. Thus, the model that results in predicted values closest to the observed values will produce the lowest MSPE. Unlike DIC, in MSPE the role of the effective number of parameters as a measure of model complexity is not clear; this suggest use of the DIC for model validation. Formally, the DIC for model *M *is defined as

where Θ_*M *_is the set of all parameters under model *M*,  is the posterior mean deviance and *p*_*M *_is the effective number of parameters, which is a measure of model complexity. The effective number of parameters is calculated by , where  is the deviance of the posterior means.

### Data sources

The above conceptual model was tested at the county level for the 2001 population of South Carolina ages 18 and over. The county specific observed numbers of hospital admissions for twelve adult ACSCs for the state of South Carolina were obtained from the State Inpatient Database (SID) for South Carolina. The nationwide numbers of hospital admissions for the reference year, year 2000, for the twelve adult ACSCs for different age- and gender-groups, were obtained from the Nationwide Inpatient Sample (NIS), with adjustment for the sampling weights. The total population in each age- and gender-group for the South Carolina state population for the reference year was obtained from the US census bureau website. The case-mix adjusted county and ACSC specific expected counts were obtained by the indirect method of standardization. In this case-mix adjustment, two important confounders were considered, age and sex, because the preliminary analysis indicated some degree of variation in these two groups for the ACSCs hospitalization rates.

The county specific data were obtained from Area Resource File (ARF) for the following manifest variables: urban-rural continuum; physicians per 1000 population; HMO penetration rate; hospital beds per 1000 population; median household income; mortality rates for liver disease, CHF, COPD and diabetes; percentage of the population that is disabled; unemployment rate; and percentage of population below the poverty level. County specific hospital visits for marker conditions, chronic conditions, elective procedures, and high-variation conditions were obtained from the SID for South Carolina.

## Results

**Figure 3:**
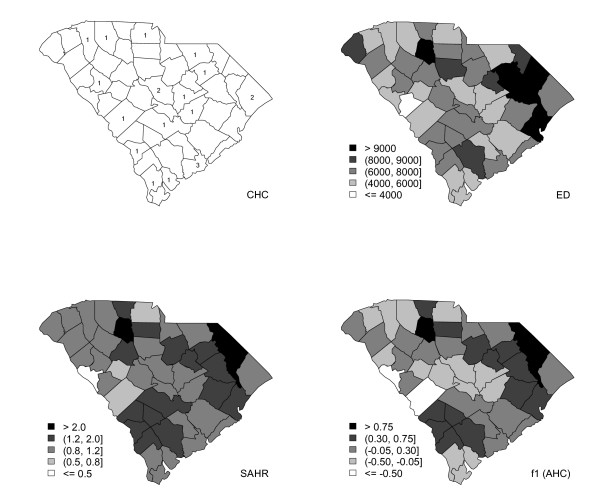
**Map for the county-wise number of CHCs in operation (top-left), and thematic maps of ED admissions (top-right), standardized ACSC hospital visit rates (bottom-left), and endogenous variable, access to health care (bottom-right)**.

The state of South Carolina has forty-six counties (i.e., *n *= 46) with various degrees of racial and economic diversity. It has twenty federally-funded community health centers (CHCs); county-wise numbers are given in the top-left of Figure 3. CHCs are widely regarded as easily accessible primary health care centers for economically disadvantaged populations. Charleston County in the east has the largest number of CHCs. The thematic map for the number of emergency department (ED) visits in 2001 is given in the top-right of Figure 3. Standardized ACSC hospitalization rates (SAHR) are given in the bottom-left of Figure 3. For the presentation in Figure 3, observed and expected ACSCs are obtained using the rates of the combined twelve adult ACSCs; this approach is used in most research that uses the ACSC indicator. The highest SAHRs are observed in the northeast region, constituted by Marlboro, Dillon and Marion counties, and Union County in the north; the lowest SAHR is observed for McCormick County in the west.

The DIC for the current model (Model3) is 8407.14, with the effective number of parameters 121.96. To validate the current model, we have also fitted two other models and observe their DIC values. The DIC values and the values for the effective number of parameters for each model are presented in Table 1. In Model1, no spatial dependence was assumed for the endogenous and exogenous factors. Model2 and Model3 considered spatial dependence for all factors. The difference lay in Model2, where flat normal priors were assigned to all the *χ *'s in (3), whereas in Model3, the prior distributions for *χ *'s are: *χ*_1 _~ *U*(0, 10), *χ*_2 _~ *U*(0, 10), *χ*_3 _~ *U*(-10, 0) and *χ*_4 _~ *U*(0, 10). The minimum and maximum values for the parameters of uniform distributions in the priors were selected based on our preliminary understanding about the influence of exogenous factors on AHC. We can see an improvement in DIC value for Model3 for these prior specifications. The results presented hereafter are for Model3.

**Table 1:** The deviance information criteria (DIC) and the effective number of parameters for the competing models

Model	Effective number of parameters	DIC
Model1	71.15	8888.45
Model2	133.33	8666.82
Model3	121.96	8407.14

The thematic map of the posterior mean of the endogenous variable representing access to health care (AHC) is given in the bottom-right portion of Figure 3. The darker regions show counties with lower rates of AHC (corresponding to higher rates of hospitalization for ACSCs); lighter colors indicate higher AHC rates. We can also see a clustering pattern; there are three distinct clusters of various sizes and shapes: one in the north, one in the south, and one extended from north to east. The strong similarity between the maps of SAHR and AHC justifies using the ACSC hospitalization rate as a manifest variable for AHC. In general, the four maps in Figure 3 are quite similar.

Table 2 gives the posterior mean estimates with the 95% credible interval (CI) of factor loadings for the endogenous variable, AHC, at measurement level. Uncontrolled diabetes, hypertension and dehydration are the most significant ACSCs for the construction of AHC. Table 3 gives the posterior mean estimates with the 95% CI of factor loadings and standard deviations for the four exogenous variables: population lifestyle/SES, physician practice behavior, population tendency to use health care and disease prevalence, at the same level. In these two tables, the first column shows the name of manifest variables, and the second and third columns show the corresponding factor loading parameters and their estimates. The third and fourth columns of Table 3 show the standard deviations of the measurement models for the exogenous factors and their estimates. All the factor loadings for the latent factor population lifestyle/SES are significant since none of the estimated credible intervals include zero. Hospital bed supply and elective procedures are significant manifest variables for the construction of physician practice behavior and population tendency to use health care, respectively. For the construction of disease prevalence, disabled and mortality are significant manifest variables. The significant loading factors always have low standard deviation.

**Table 2:** Posterior mean (95% credible interval) estimates of measurement model parameters of an endogenous variable for the conceptual model for South Carolina population ages 18 and over

Manifest variable	Parameter	Estimate
Short-term diabetes complications		1
Long-term diabetes complications		1.102 (0.964, 1.265)
Uncontrolled diabetes		1.690 (1.477, 1.945)
Lower extremity amputation in diabetic patients		1.094 (0.929, 1.285)
Adult asthma		1.071 (0.935, 1.235)
Hypertension		1.559 (1.369, 1.788)
Dehydration		1.344 (1.189, 1.533)
UTI		1.075 (0.944, 1.233)
Bacterial pneumonia		0.740 (0.650, 0.849)
Angina without procedure		0.449 (0.324, 0.583)
COPD		0.852 (0.747, 0.977)
CHF		0.838 (0.742, 0.956)

**Table 3:** Posterior mean (95% credible interval) estimates of measurement model parameters of four exogenous variable for the conceptual model for South Carolina population ages 18 and over

Manifest variable	Parameter	Estimate	Parameter	Estimate
Median household income		-1.021 (-1.280, -0.806)		0.473 (0.367, 0.598)
Poverty		1.134 (0.961, 1.355)		0.160 (0.022, 0.317)
Unemployment		0.951 (0.714, 1.224)		0.578 (0.454, 0.731)
African-Americans		1		0.463 (0.364, 0.586)
Physician supply		-0.018 (-0.320, 0.289)		1.030 (0.841, 1.277)
Hospital bed supply		0.376 (0.093, 0.664)		0.955 (0.776, 1.189)
High-variation conditions		1		0.079 (0.005, 0.185)
Elective procedures		-4.682 (-8.908, -2.353)		0.406 (0.515, 0.611)
HMO penetration		-0.548 (-2.330, 1.078)		1.020 (0.829, 1.268)
Rural residence		1		1.043 (0.848, 1.298)
Disability		0.533 (0.267, 0.800)		0.877 (0.709, 1.095)
Mortality		0.774 (0.572, 0.979)		0.662 (0.534, 0.823)
Marker conditions		0.191 (-0.112, 0.494)		1.009 (0.823, 1.252)
Chronic conditions		1		0.133 (0.005, 0.233)

The posterior means with 95% credible intervals for all parameters in the structural equation model are given in Table 4. All of the regression coefficients are significant. Among them, the latent factors (population lifestyle/SES, physician practice behavior and disease prevalence) contribute positively to the lack of AHC. The other latent factor, population tendency to use health care, contributes positively to the increase of AHC. The spatial correlation for the latent factor for AHC is close to one, indicating strong similarities among the spatial distributions of ACSCs. The spatial correlations for the other latent factors are moderate.

**Table 4:** Posterior mean (95% credible interval) estimates of structural equation model parameters of conceptual model for South Carolina population ages 18 and over

Parameter	Estimate	Parameter	Estimate
*χ*_1_	0.126 (0.022, 0.252)	*a*_1_	1.660 (1.226, 2.193)
*χ*_2_	0.129 (0.005, 0.333)	*a*_3_	1.927 (1.538, 2.403)
*χ*_3_	-0.707 (-2.248, -0.033)	*a*_6_	0.148 (0.027, 0.367)
*χ*_4_	0.131 (0.006, 0.323)	*a*_10_	0.232 (0.024, 0.461)
*a*_2_	0.634 (0.053, 1.248)	*σ*_*κ*_	0.419 (0.308, 0.557)
*a*_4_	0.162 (0.048, 0.345)	*ρ*_*κ*_	0.902 (0.684, 0.993)
*a*_5_	-0.416 (-0.771, -0.171)		0.766 (0.348, 0.977)
*a*_7_	-0.218 (-0.634, 0.407)		0.552 (0.069, 0.928)
*a*_8_	1.654 (-0.045, 2.816)		0.501 (0.032, 0.943)
*a*_9_	-0.066 (-0.307, 0.157)		0.502 (0.027, 0.959)

**Figure 4:**
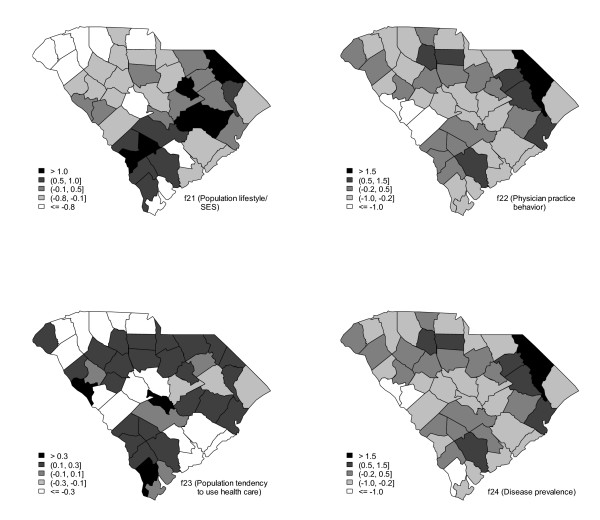
**Thematic maps of four exogenous variables, disease prevalence (top-left), population tendency to use health care resources (top-right), physician practice behaviors (bottom-left), and population lifestyle (bottom-right)**.

Figure 4 displays the thematic maps of four exogenous variables: population lifestyle/SES, physician practice behavior, population tendency to use health care, and disease prevalence. In all of these four maps, darker counties indicate unhealthful lifestyle/SES, inadequate physician practice behavior, a low tendency to use health care resources, and high rates of disease prevalence.

**Figure 5:**
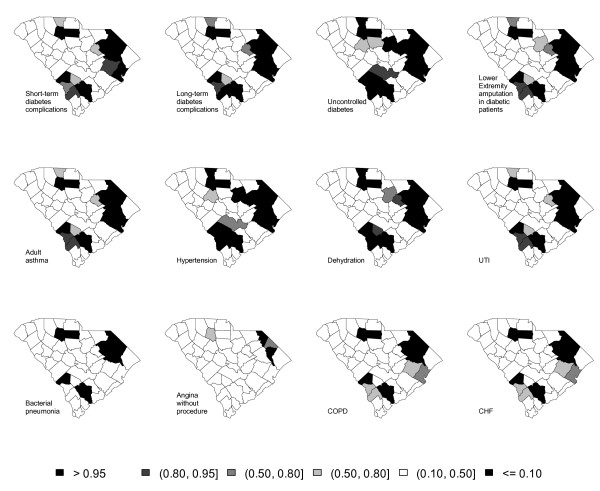
**Thematic maps of exceedance probability of twelve adult ACSC hospital visits**.

**Figure 6:**
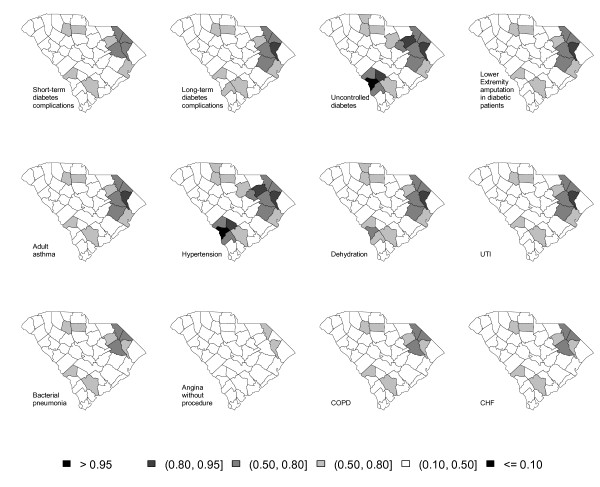
**Thematic maps of *R*_*i*_, *i *= 1,...,46 of twelve adult ACSC hospital visits**.

Figure 5 displays the exceedance probability for each ACSC where *c *= 1.5. The value for the threshold is chosen arbitrarily. A darker color indicates excess risk of ACSC admission. The maps tend to show a clustering pattern. The largest clusters are obtained for uncontrolled diabetes and hypertension; factor loading estimates for these two ACSCs were 1.690 and 1.559, respectively. For these two ACSCs, one cluster in the east extends to the state's center; one appears in the north and one in the south. Similar clustering is also shown for short-term diabetes complications, long-term diabetes complications, lower extremity amputation in diabetic patients, adult asthma, dehydration, UTI, bacterial pneumonia, COPD and CHF. The smallest cluster is obtained for angina without procedure, for which the loading factor estimate was 0.4434. Figure 6 displays the maps for *R *after using a cluster detection tool. Figure 6 signals similar clustering patterns as Figure 5; the tendency is for counties with the highest exceedence probabilities in Figure 5 to have slightly weaker signals in Figure 6.

## Discussion

By using generalized spatial structural equation modeling, we attempted to identify how population lifestyle/SES, physician practice behaviors, population tendency to use health care resources, and disease prevalence are associated with access to primary health care, as measured by hospitalizations for ACSCs. We observed that counties having low access to primary health care also have unhealthful lifestyles, inadequate physician practice behaviors, a low tendency to use health care and high rates of disease prevalence.

The overall strength of this research lies in the importance of showing the geographical distributions (i.e., maps) of each latent factor: access to health care, population lifestyle/SES, physician practice behaviors, population tendency to use health care resources, and disease prevalence. Because of the unobservable nature of these factors, we used a multivariate spatial structural equation modeling approach. To measure the underlying factor for AHC, we used all of the ACSCs individually, an approach that retains useful information in the modeling. By doing this for South Carolina hospital discharge data for the year 2001, we confirmed a similar spatial distribution of AHC and ED visits. These two maps also have strong resemblance to the spatial distribution of CHC locations. Counties that had no CHC had the least access to primary health care and more ED visits. This finding is consistent with the limited relevant research literature on the effectiveness of CHCs for improving access [35-37] and a large body of research on factors associated with ED visits. The CHC finding has substantial policy relevance, as it is often anticipated that CHCs will be located in counties having the greatest need to improve the accessibility or quality of primary health care. The results suggest that the counties that had the lowest estimated levels of access to health care might benefit from having CHCs, which can reduce rates of expensive ED utilization.

This research also proposed to find the clusters of counties with excess risk for ACSC hospitalization, utilizing a cluster detection tool. In the computation of exceedance probability, we set the threshold value to 1.5. Higher threshold values could also be of interest (e.g., 3) to find high-risk counties. The result would locate counties where the accessibility or quality of primary health care may be particularly inadequate; these counties would be especially appropriate for targeted policy actions to enhance primary health care. This result illustrates the practical value of identifying spatial clusters with a relatively high likelihood of having barriers to primary health care.

Access to health care can also be viewed as a dynamic process, i.e. besides the spatial dimensionality, it may also vary temporally. In our future work, we propose to extend the multivariate spatial structural equation models to space-time data, since health care data are now regularly available for repeated years at the level of geographical units. The space-time analysis will show the spatial and temporal distribution of those latent factors, and will locate clusters of under-served regions that are persistent over time. The extension to space-time analysis will be useful for examining effects of policy changes designed to improve access to primary health care. It will also be useful for examining effects of state reductions in health care for vulnerable populations in the United States Medicaid program.

## Competing interests

The authors declare that they have no competing interests.

## Authors' contributions

MMH conceived the paper, performed the statistical analysis and drafted the manuscript. JNL provided health services research expertise, assisted in the development of the conceptual model, and contributed to the manuscript. Both authors read and approved the final manuscript.
